# Gender differences in the association between pan-immune-inflammation value and probable depression: A cross-sectional study

**DOI:** 10.1371/journal.pone.0339348

**Published:** 2025-12-30

**Authors:** Dongdong Yu, Ting Cheng, Yue Yang, Xingying Qiu, Geng Li, Li Zhou, Zehuai Wen

**Affiliations:** 1 Second Clinical College of Guangzhou University of Chinese Medicine, Guangzhou, Guangdong, China; 2 First Affiliated Hospital of Anhui University of Chinese Medicine, Hefei, Anhui, China; 3 Center for Clinical Research of Guangdong Provincial Hospital of Chinese Medicine, Second Affiliated Hospital of Guangzhou University of Chinese Medicine, Guangzhou, Guangdong, China; 4 Science and Technology Innovation Center of Guangzhou University of Chinese Medicine, Guangzhou, Guangdong, China; NED University of Engineering and Technology, PAKISTAN

## Abstract

This study explored the non-linear relationship between pan-immune inflammation value (PIV) and probable depression using NHANES data (2005–2018, n = 27,049). Restricted cubic spline analysis identified an optimal PIV cutoff (5.74). Weighted logistic regression revealed that individuals with PIV > 5.74 had 14% higher risk of probable depression (OR=1.14, 95% CI:1.02–1.28). Gender-stratified analyses showed significant PIV and probable depression associations in females (OR=1.20, 95% CI:1.03–1.40) but not in males. In males, higher PIV interacted with age, education, and alcohol consumption to influence probable depression risk (interaction *P* < 0.05), whereas no such interactions occurred in females. The findings suggest PIV may serve as a sex-specific biomarker for probable depression risk, with more stable associations in females. Further research is needed to elucidate the mechanisms underlying these gender differences and validate these findings before any clinical application.

## Introduction

Major depressive disorder (MDD) represents a significant global health challenge, ranking as a leading cause of disability worldwide and imposing a substantial emotional and economic burden on individuals, families, and healthcare systems [1–4]. The complexity of its etiology necessitates the identification of objective, modifiable risk factors to enhance early detection and inform personalized prevention strategies.

In recent years, the neuroinflammatory hypothesis of depression has gained widespread attention, suggesting that immune system dysregulation plays a critical role in its pathophysiology [5]. A large body of evidence indicates that individuals with depression exhibit increased levels of pro-inflammatory cytokines and activation of microglia [6–9]. Consequently, various peripheral blood cell indices and leukocyte-derived ratios (LDRs) have been explored as accessible and cost-effective biomarkers. Studies have linked indices such as the platelet-to-lymphocyte ratio (PLR) [10,11], neutrophil-to-lymphocyte ratio (NLR) [12,13], lymphocyte-to-monocyte ratio (LMR) [14], systemic immune-inflammation index (SII) [15–17], and systemic inflammatory response index (SIRI) [18] to depression risk. The pan-immune-inflammation value (PIV), calculated from neutrophils, platelets, monocytes, and lymphocytes, is a newer, more comprehensive composite biomarker [19,20]. In contrast to simpler two- or three-cell ratios, PIV may offer a more nuanced reflection of the complex interplay between innate and adaptive immune pathways. PIV has been associated with a wide range of non-communicable diseases, including cardiovascular and metabolic conditions such as metabolic dysfunction-associated steatotic liver disease (MASLD) [21–28], but its value in predicting depression risk remains unconfirmed.

Clinical and preclinical studies suggest that depression shows significant gender differences in etiology, pathophysiology, and immune function, with women having approximately twice the risk of depression as men [29–32]. However, research on whether biological sex modifies the relationship between systemic inflammation and depression remains limited [13,29,33]. Therefore, this study was designed to be explicitly hypothesis-driven. We hypothesized that the association between PIV and probable depression is stronger and more stable in women, who exhibit distinct hormonal and immunological profiles, compared to men. Using a large, nationally representative U.S. sample, we aimed to explore the association between PIV and probable depression, with a specific focus on gender differences, to provide new perspectives for personalized depression risk assessment.

## Methods

### Study population

This study extracted data from seven continuous cycles of the National Health and Nutrition Examination Survey (NHANES) between 2005 and 2018. NHANES is a national program designed to evaluate the health and nutritional status of the U.S. population using a complex, multistage probability sampling design. The survey protocol was approved by the National Center for Health Statistics (NCHS) ethics review board, and all participants provided informed consent. As this is a secondary analysis of de-identified public data, no additional ethical approval was required.

**Fig 1:**
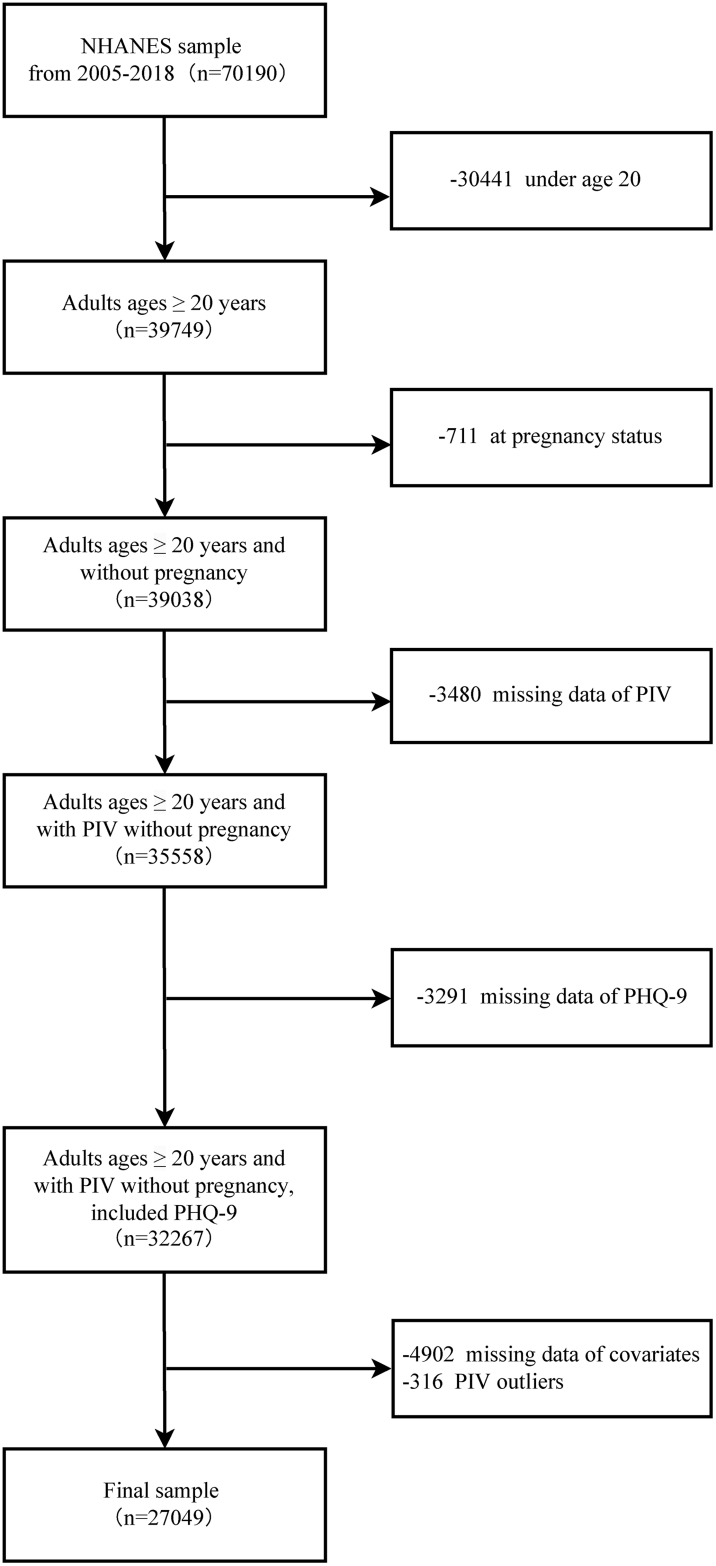
Flowchart of participant selection from NHANES 2005–2018.

From an initial sample of 70,190 individuals, we excluded participants aged < 20 years (n = 30,441) and those who were pregnant (n = 711). We further excluded individuals with missing data for the Patient Health Questionnaire-9 (PHQ-9) (n = 3,291), PIV components (neutrophil, platelet, lymphocyte, or monocyte counts) (n = 3,480), or any covariates (n = 4,902). Finally, we excluded 316 participants with extreme PIV values (beyond ±3 standard deviations from the mean) to minimize the influence of biologically implausible outliers, a standard practice in large-scale epidemiological studies [34]. The final analytical sample comprised 27,049 participants (Fig 1). The data used in this study can be freely accessed through the following website: https://wwwn.cdc.gov/nchs/nhanes/Default.aspx.

### Definition of PIV

Peripheral blood cell counts were measured using a Coulter® DxH 800 Analyzer. PIV was calculated using the established formula: PIV = (neutrophil count × platelet count × monocyte count)/ lymphocyte count [21,22].

### Assessment of probable depression

The primary outcome was probable depression, assessed using the PHQ-9, a validated 9-item screening tool [35]. The PHQ-9 was administered by trained interviewers using the computer-assisted personal interview (CAPI) system. Consistent with established literature, a total score ≥10 was used to define a case of current probable depression [15]. This term is used throughout the manuscript to clarify that the outcome is based on a screening tool rather than a formal clinical diagnosis of MDD.

### Covariates

We included all possible factors that could influence the relationship between PIV and depression risk [36]. Key demographic information included survey cycle, age, gender, race, education level, marital status, and healthcare insurance status. Self-reported smoking and alcohol consumption statuses are binary variables, with “no” defined as having never smoked or drunk, and “yes” defined as having ever smoked or drunk. Participants with < 100 lifetime cigarettes were classified as nonsmokers (“no”); all others as smokers (“yes”, including former/current). Individuals reporting < 12 alcoholic drinks in their lifetime were classified as nondrinkers (“no”); other respondents (including former/current drinkers) as drinkers (“yes”). Body mass index (BMI) was calculated based on measurements from the examination center, with categories of <18.5, 18.5–25, 25–30, and >30 kg/m^2^. Abdominal obesity was defined as a waist circumference ≥102 cm for men and ≥88 cm for women [37]. The poverty income ratio (PIR) refers to the ratio of household income to the federal poverty line; lower values indicate higher poverty levels [38]. Self-reported physical activity included walking, cycling, household/gardening work, strength training, and work or leisure activities; individuals engaging in any of these activities were classified as “physically active”.

In all survey cycles, hypertension was defined if any of the following criteria were met: (1) self-reported medical history, (2) informed by a healthcare provider, (3) currently using antihypertensive medications, (4) average systolic blood pressure (SBP) ≥ 140 mmHg, and/or average diastolic blood pressure (DBP) ≥ 90 mmHg. Diabetes was defined by meeting any of the following criteria: (1) informed by a healthcare provider, (2) HbA1c ≥ 6.5%, (3) fasting blood glucose ≥ 7.0 mmol/L, (4) random blood glucose ≥ 11.1 mmol/L, (5) 2-hour oral glucose tolerance test (OGTT) blood glucose ≥ 11.1 mmol/L, (6) currently using insulin or oral hypoglycemic medications. The estimated glomerular filtration rate (eGFR) was calculated using the chronic kidney disease (CKD) EPI 2021 equation, with eGFR ≤ 60 mL/min/1.73 m^2^ defined as CKD. Covariates also included self-reported cardiovascular diseases (congestive heart failure, coronary heart disease, angina, or myocardial infarction), stroke (yes/no), cancer, and the use of antidepressant prescription medications.

### Statistical analysis

Missing data were handled using a complete-case analysis approach, which is a common method in NHANES-based studies. Participants with missing values for any variable of interest were excluded from the analysis, as described in the exclusion criteria. Considering the complex design of NHANES and following the NHANES analysis and reporting guidelines, weighted sample analyses were performed using survey weights. Since PIV concentrations were highly skewed, log transformation was applied to the standardized data during analysis to approximate a normal distribution. The Chi-square test or t-test was used to assess the baseline characteristics of participants. Continuous variable is expressed as mean ± standard deviation (SD) with 95% confidence intervals (CI), and categorical variables is presented as percentages with 95% CI. Standardized mean differences (SMDs) were calculated to assess the magnitude of baseline differences [39].

The non-linear relationship between PIV and probable depression was examined using a weighted restricted cubic spline (RCS) model [40], adjusted for all covariates. The PIV value at the nadir (lowest risk) of the U-shaped RCS curve was identified and used as a data-driven cutoff to dichotomize participants into lower-PIV and higher-PIV groups. An analysis using PIV as a continuous variable was also included in the sensitivity analysis. Weighted logistic regression models were employed to assess the association between PIV (as both a continuous and a binary variable) and depression, with all analyses conducted following the guidelines provided by the NCHS—guidelines that recommend using Taylor Series Linearization for variance estimation in the context of the complex NHANES survey design—and reporting odds ratio (OR) and its 95% CI. We assessed for multicollinearity among covariates using the Variance Inflation Factor (VIF). All VIF values were less than 5, indicating that multicollinearity was not a significant concern in our models. The analysis was conducted on the overall sample, and stratified analyses by gender were also performed.

Three adjusted models were constructed to verify the robustness of the results: the crude model, with no adjustments; model 1, which adjusted for age, gender, race, education level, PIR, smoking, drinking, and health insurance; model 2, which further adjusted for central obesity, BMI, and physical activity; and model 3, which further adjusted for the history of antidepressant use, diabetes, hypertension, CVD, CKD, and cancer. Sensitivity analyses were performed by excluding individuals on antidepressant therapy and re-running all models (crude, Model 1, Model 2, and Model 3). Additionally, exploratory interaction analyses between age, education level, and alcohol consumption status with PIV groups were performed to investigate how these factors may modify the association between PIV and depression risk. These were not pre-specified hypotheses and should be interpreted with caution. Statistical significance was set at a two-tailed *P* value of < 0.05. All statistical analyses were performed using R 4.4.0 software.

## Results

### Characteristics of the study population

**Fig 2:**
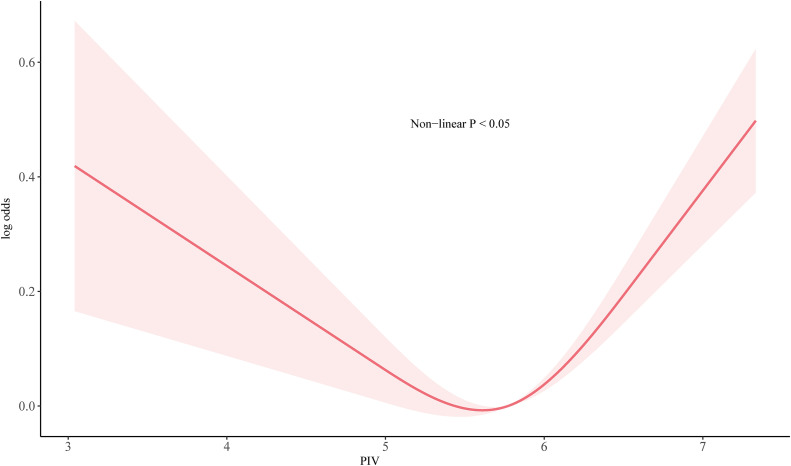
Restricted cubic spline analysis of the association between Pan-Immune-Inflammation Value (PIV) and probable depression.

This study included 27,049 participants. Based on the cutoff value for PIV determined by RCS, participants were divided into a higher PIV group (PIV > 5.74, n = 9278) and a lower PIV group (PIV ≤ 5.74, n = 17771) (Fig 2). Compared to the lower PIV group, participants in the higher PIV group were older, had a higher proportion of females, had more individuals living alone, had a higher smoking rate, more individuals in poverty, less physical activity, more abdominal obesity, and a higher proportion of comorbidities such as diabetes, stroke, cancer, CKD, and cardiovascular disease. The depression prevalence was also higher in the higher PIV group. Baseline characteristics are presented in Table 1. While many differences were statistically significant due to the large sample size, the SMDs indicated that the baseline balance was excellent. The vast majority of variables had negligible differences (SMD < 0.1). The only variable with a medium difference was race/ethnicity (SMD = 0.235), with the higher PIV group having a larger proportion of Non-Hispanic White participants. Minor differences (SMD 0.1–0.2) were observed for education, smoking, BMI, central obesity, and hypertension. Overall, the two groups were well-balanced. More detailed characteristics of the participants are shown in Table 1.

**Table 1:** Baseline characteristics of participants by PIV group.

Variable	Total (n = 27049)	Higher PIV (n = 9278)	Lower PIV(n = 17771)	*P*	*SMDs*
Year				**< 0.001**	0.187
2005-2006	14.22 (12.45, 15.99)	17.35 (15.11, 19.58)	12.45 (11.04, 13.87)		
2007-2008	13.99 (12.21, 15.76)	14.90 (12.70, 17.09)	13.47 (12.01, 14.93)		
2009-2010	13.94 (12.25, 15.62)	12.63 (10.99, 14.26)	14.68 (13.08, 16.27)		
2011-2012	14.49 (12.52, 16.47)	11.82 (9.61, 14.04)	16.00 (14.07, 17.93)		
2013-2014	15.25 (13.52, 16.98)	15.20 (13.56, 16.84)	15.28 (13.43, 17.13)		
2015-2016	15.12 (13.28, 16.96)	15.76 (13.41, 18.11)	14.75 (13.21, 16.30)		
2017-2018	13.00 (12.14, 13.86)	12.34 (11.14, 13.54)	13.37 (12.26, 14.48)		
Age				**< 0.001**	0.091
< 60	75.18 (71.75, 78.61)	72.65 (71.09, 74.20)	76.62 (75.48, 77.75)		
≥60	24.82 (23.18, 26.46)	27.35 (25.80, 28.91)	23.38 (22.25, 24.52)		
Sex				0.910	0.002
Male	49.41 (47.13, 51.68)	49.48 (48.19, 50.76)	49.37 (48.45, 50.29)		
Female	50.59 (48.21, 52.97)	50.52 (49.24, 51.81)	50.63 (49.71, 51.55)		
Race/Ethnicity				**< 0.001**	0.235
Non-Hispanic White	69.74 (64.46, 75.01)	75.20 (72.93, 77.46)	66.65 (64.00, 69.30)		
Non-Hispanic Black	10.14 (9.09, 11.18)	6.20 (5.31, 7.09)	12.36 (10.84, 13.89)		
Mexican American	8.08 (6.89, 9.27)	7.72 (6.38, 9.07)	8.28 (6.97, 9.60)		
Other	12.05 (11.15, 12.95)	10.88 (9.74, 12.02)	12.70 (11.53, 13.88)		
Marital status				**< 0.001**	0.075
Married or partner	64.59 (60.88,68.31)	62.28 (60.73, 63.83)	65.90 (64.50, 67.29)		
Alone	35.41 (34.03, 36.78)	37.72 (36.17, 39.27)	34.10 (32.71, 35.50)		
Education				**< 0.001**	0.11
Less than high school	4.60 (4.15, 5.05)	4.28 (3.69, 4.87)	4.78 (4.27, 5.28)		
High school or equivalent	32.96 (30.83, 35.09)	36.26 (34.28, 38.23)	31.10 (29.54, 32.65)		
College or above	62.44 (59.04, 65.85)	59.46 (57.35, 61.58)	64.13 (62.33, 65.92)		
Health insurance				0.370	0.013
No	17.02 (15.96, 18.08)	17.34 (16.05, 18.62)	16.84 (15.71, 17.98)		
Yes	82.98 (78.74, 87.22)	82.66 (81.38, 83.95)	83.16 (82.02, 84.29)		
PIR				**0.010**	0.058
< 1.3	20.12 (18.92, 21.32)	21.08 (19.53, 22.63)	19.58 (18.36, 20.79)		
1.3-3.5	35.52 (33.59, 37.44)	36.33 (34.91, 37.75)	35.05 (33.62, 36.49)		
> 3.5	44.37 (41.24, 47.49)	42.59 (40.41, 44.77)	45.37 (43.34, 47.40)		
Smoke				**< 0.001**	0.17
No	54.97 (52.47, 57.47)	49.57 (47.85, 51.28)	58.02 (56.83, 59.22)		
Yes	45.03 (42.51, 47.55)	50.43 (48.72, 52.15)	41.98 (40.78, 43.17)		
Alcohol				**< 0.001**	0.063
No	10.23 (9.26, 11.19)	9.02 (8.04, 10.00)	10.91 (9.94, 11.87)		
Yes	89.77 (85.60, 93.95)	90.98 (90.00, 91.96)	89.09 (88.13, 90.06)		
BMI				**< 0.001**	0.185
18.5-25	28.19 (26.59, 29.79)	24.85 (23.65, 26.06)	30.08 (28.83, 31.32)		
< 18.5	1.46 (1.26, 1.66)	1.35 (1.04, 1.67)	1.52 (1.28, 1.76)		
25-30	33.09 (31.31, 34.87)	30.91 (29.67, 32.15)	34.32 (33.20, 35.44)		
> 30	37.26 (35.31, 39.20)	42.88 (41.51, 44.24)	34.08 (32.81, 35.35)		
Central obesity				**< 0.001**	0.181
No	44.02 (41.84, 46.20)	38.33 (36.91, 39.75)	47.24 (45.77, 48.70)		
Yes	55.98 (53.01, 58.95)	61.67 (60.25, 63.09)	52.76 (51.30, 54.23)		
Physical activity				**< 0.001**	0.096
No	19.64 (18.48, 20.80)	22.09 (20.79, 23.39)	18.26 (17.32, 19.19)		
Yes	80.36 (76.54, 84.18)	77.91 (76.61, 79.21)	81.74 (80.81, 82.68)		
Hypertension				**< 0.001**	0.168
No	63.04 (60.05, 66.04)	57.86 (56.34, 59.39)	65.97 (64.80, 67.14)		
Yes	36.96 (35.01, 38.90)	42.14 (40.61, 43.66)	34.03 (32.86, 35.20)		
Diabetes				**< 0.001**	0.124
No	86.45 (82.43, 90.47)	83.70 (82.71, 84.70)	88.01 (87.37, 88.64)		
Yes	13.55 (12.75, 14.35)	16.30 (15.30, 17.29)	11.99 (11.36, 12.63)		
Stroke				**< 0.001**	0.071
No	97.37 (92.98, 101.76)	96.62 (96.18, 97.06)	97.79 (97.55, 98.03)		
Yes	2.63 (2.39, 2.87)	3.38 (2.94, 3.82)	2.21 (1.97, 2.45)		
Cancer				**< 0.001**	0.065
No	90.34 (86.32, 94.37)	89.10 (88.15, 90.04)	91.05 (90.48, 91.61)		
Yes	9.66 (8.97, 10.34)	10.90 (9.96, 11.85)	8.95 (8.39, 9.52)		
CVD				**< 0.001**	0.087
No	93.75 (89.52, 97.98)	92.37 (91.69, 93.05)	94.53 (94.10, 94.96)		
Yes	6.25 (5.76, 6.73)	7.63 (6.95, 8.31)	5.47 (5.04, 5.90)		
CKD				**< 0.001**	0.088
No	94.78 (90.47, 99.09)	93.49 (92.89, 94.10)	95.50 (95.11, 95.90)		
Yes	5.22 (4.83, 5.61)	6.51 (5.90, 7.11)	4.50 (4.10, 4.89)		
Antidepressant medication				0.080	0.032
No	91.44 (87.35, 95.53)	90.87 (90.10, 91.63)	91.76 (91.04, 92.48)		
Yes	8.56 (7.86, 9.26)	9.13 (8.37, 9.90)	8.24 (7.52, 8.96)		
Depression				**< 0.001**	0.075
No	92.62 (88.40, 96.85)	91.35 (90.63, 92.08)	93.34 (92.81, 93.86)		
Yes	7.38 (6.82, 7.94)	8.65 (7.92, 9.37)	6.66 (6.14, 7.19)		

Data are presented as mean ± SD or n (%). Abbreviations are defined as follows: BMI, body mass index; CKD, chronic kidney disease; CVD, cardiovascular disease; PIR, poverty income ratio; PIV, pan-immune-inflammation value; SMD, standardized mean difference. *P* < 0.05. SMD < 0.1 indicates a negligible difference, 0.1–0.2 small difference, 0.2–0.5 medium difference, 0.5–0.8 large difference, and >0.8 very large difference.

The curve demonstrates a U-shaped relationship with a nadir at PIV = 5.74. Both lower and higher PIV values are associated with increased odds of probable depression. Shaded area represents the 95% confidence interval.

### Associations of the PIV with probable depression

Weighted logistic regression analysis revealed a significant positive association between PIV and probable depression. In the fully adjusted Model 3, when PIV was treated as a continuous variable, each unit increase was associated with an 11% higher odds of depression (OR=1.11, 95% CI: 1.00–1.24, P = 0.041). As a categorical variable, the high PIV group had 14% higher odds than the low PIV group (OR=1.14, 95% CI: 1.02–1.28, P = 0.021). The findings were consistent across all unadjusted and partially adjusted models (all *P* < 0.05, see Table 2).

**Table 2:** Association between PIV and probable depression.

Character	Crude model		Model 1		Model 2		Model 3	
	OR (95%CI)	*P*	OR (95%CI)	*P*	OR (95%CI)	*P*	OR (95%CI)	*P*
PIV (continuous)	1.25 (1.13, 1.39)	**<0.001**	1.19 (1.08, 1.32)	**<0.001**	1.14 (1.03, 1.26)	**0.011**	1.11 (1.00, 1.24)	**0.041**
PIV category								
Lower PIV	ref		ref		ref		ref	
Higher PIV	1.33 (1.19, 1.48)	**<0.001**	1.24 (1.11, 1.38)	**<0.001**	1.18 (1.06, 1.31)	**0.003**	1.14 (1.02, 1.28)	**0.021**

PIV: pan-immune inflammation value; BMI: body mass index; PIR: poverty income ratio; CVD: cardiovascular disease; CKD: chronic kidney disease; Bold indicates *P* < 0.05; ref: reference level/category.

Crude model: adjusted for none.

Model 1: Crude model + adjusted for age, sex, race, education, PIR, smoke, alcohol, health insurance.

Model 2: Model 1 + adjusted for central obesity, BMI, and physical activity.

Model 3: Model 2 + adjusted for antidepressant use, diabetes, hypertension, CVD, CKD, cancer, and stroke.

A sensitivity analysis excluding antidepressant users confirmed the robustness of these results. The association remained significant among non-users (fully adjusted OR=1.15 (1.00–1.33), P = 0.046), with a consistent pattern that reinforces the validity of our findings (see S1 Table).

### Gender differences in the associations between PIV with probable depression

We conducted gender-stratified weighted logistic regression analyses to explore the relationship between PIV and probable depression (Table 3 and S2 Table). In the crude model, higher PIV levels were significantly associated with increased probable depression risk in both females (continuous: OR = 1.29, 95% CI: 1.14–1.46, *P* < 0.001; categorical: OR = 1.41, 95% CI: 1.21–1.63, *P* < 0.001) and males (continuous: OR = 1.20, 95% CI: 1.03–1.41, *P* = 0.021; categorical: OR = 1.21, 95% CI: 1.02–1.44, *P* = 0.032).

**Table 3:** Sex-specific associations between PIV and probable depression.

Character	Female OR(95%CI)	*P*	Male OR(95%CI)	*P*
PIV (continuous)	1.13 (1.00, 1.27)	0.055	1.09 (0.92, 1.29)	0.323
PIV category				
Lower PIV	ref		ref	
Higher PIV	1.20 (1.03, 1.40)	**0.018**	1.05 (0.87, 1.26)	0.622

PIV: pan-immune inflammation value; BMI: body mass index; PIR: poverty income ratio; CVD: cardiovascular disease; CKD: chronic kidney disease; Bold indicates P < 0.05; ref: reference level/category. Model: adjusted for age, sex, race, education, PIR, smoke, alcohol, health insurance, central obesity, BMI, and physical activity, antidepressant use, diabetes, hypertension, CVD, CKD, cancer, and stroke.

However, after adjusting for covariates, the association remained significant only in females. In the fully adjusted Model 3, females in the higher PIV group had 20% higher odds of probable depression (OR = 1.20, 95% CI: 1.03–1.40, *P* = 0.018), while PIV as a continuous variable showed a marginally significant association (OR = 1.13, 95% CI: 1.00–1.27, *P* = 0.055). In contrast, no significant association was observed in males (categorical: OR = 1.05, 95% CI: 0.87–1.26, *P* = 0.622; continuous: OR = 1.09, 95% CI: 0.92–1.29, *P* = 0.323).

### Interaction analysis of the age/education/ alcohol with PIV among different gender

We further examined whether the association between PIV and probable depression varied by demographic and lifestyle factors (Tables 4 and 5). In males, nominally significant interactions (uncorrected *P* < 0.05) were observed between higher PIV levels and age (*P* for interaction = 0.018), education (*P* for interaction = 0.011), and alcohol consumption (*P* for interaction = 0.018). After applying Bonferroni correction for multiple testing (corrected *P* = 0.017), only the education interaction remained statistically significant (Bonferroni-corrected *P* for interaction = 0.011), while the age and alcohol interactions did not survive correction (both Bonferroni-corrected *P* for interaction = 0.017).

**Table 4:** Modification of the association between PIV and probable depression by age, education, and alcohol consumption in the female subgroup.

Character	Lower PIV* vs Higher PIV	*P*	*P* for interaction
**Female**			
Age			0.586
< 60	1.39 (1.18, 1.63)	<0.001	
≥60	1.51 (1.13, 2.02)	0.006	
Education			0.578
Less than high school	1.25 (0.82, 1.91)	0.303	
High school or equivalent	1.30 (1.08, 1.56)	0.006	
College or above	1.46 (1.18, 1.80)	<0.001	
Alcohol			0.678
No	1.29 (0.87, 1.93)	0.206	
Yes	1.41 (1.21, 1.65)	<0.001	

* Lower PIV is a reference group. PIV: Pan-immune-inflammation.

**Table 5:** Modification of the association between PIV and probable depression by age, education, and alcohol consumption in the male subgroup.

Character	Lower PIV* vs Higher PIV	*P*	*P* for interaction
**Male**			
Age			0.018
< 60	1.73 (1.27, 2.37)	<0.001	
≥60	1.11 (0.90, 1.36)	0.322	
Education			0.011
Less than high school	1.68 (1.06, 2.66)	0.029	
High school or equivalent	0.87 (0.67, 1.13)	0.306	
College or above	1.46 (1.09, 1.94)	0.011	
Alcohol			0.018
No	0.52 (0.25, 1.11)	0.091	
Yes	1.24 (1.04, 1.49)	0.016	

* Lower PIV is a reference group. PIV: Pan-immune-inflammation.

Specifically, for age stratification in males, higher PIV was associated with a 73% increased odds of probable depression among those aged <60 years (OR = 1.73, 95% CI: 1.27–2.37, *P* < 0.001), whereas no significant association was observed in those aged ≥60 years (OR = 1.11, 95% CI: 0.90–1.36, *P* = 0.322). For education stratification in males, higher PIV was associated with increased probable depression risk among those with less than high school education (OR = 1.68, 95% CI: 1.06–2.66, *P* = 0.029) and college or above education (OR = 1.46, 95% CI: 1.09–1.94, *P* = 0.011), but not among those with high school or equivalent education (OR = 0.87, 95% CI: 0.67–1.13, *P* = 0.306). For alcohol consumption stratification in males, higher PIV was significantly associated with probable depression risk among drinkers (OR = 1.24, 95% CI: 1.04–1.49, *P* = 0.016) but not among non-drinkers (OR = 0.52, 95% CI: 0.25–1.11, *P* = 0.091).

In contrast, no significant interactions were observed in females. For age stratification in females, the ORs were 1.39 (95% CI: 1.18–1.63, *P* < 0.001) for < 60 years and 1.51 (95% CI: 1.13–2.02, *P* = 0.006) for ≥60 years (*P* for interaction = 0.586). For education stratification in females, the ORs were 1.25 (95% CI: 0.82–1.91, *P* = 0.303) for less than high school, 1.30 (95% CI: 1.08–1.56, *P* = 0.006) for high school or equivalent, and 1.46 (95% CI: 1.18–1.80, *P* < 0.001) for college or above (*P* for interaction = 0.578). For alcohol consumption stratification in females, the ORs were 1.29 (95% CI: 0.87–1.93, *P* = 0.206) for non-drinkers and 1.41 (95% CI: 1.21–1.65, *P* < 0.001) for drinkers (*P* for interaction = 0.678).

## Discussion

This large, nationally representative study is the first to report a significant, non-linear U-shaped association between PIV and probable depression in the U.S. population. Our findings support our primary hypothesis, revealing a more robust and stable association in females, whereas in males, the relationship was moderated by age, education, and alcohol use. These results highlight PIV as a potential sex-specific biomarker for depression risk and underscore the importance of considering gender in immuno-psychiatry research [41].

Our findings align with and extend previous research linking simpler inflammatory indices like NLR and SII to depression [12,13,15–17]. The key advantage of PIV is its integration of four immune cell types, potentially offering a more comprehensive assessment of systemic inflammation [19,20]. The U-shaped relationship is a key finding, suggesting that both low and high PIV levels are associated with increased depression risk. This non-linear pattern, which requires replication, highlights the potential importance of immune balance for mental health, rather than a simple “lower is better” inflammatory state.

The most striking finding was the pronounced gender difference. The stable association in females, independent of moderators, contrasts sharply with the context-dependent association in males. This aligns with known sex differences in immune function, neurobiology, and depression prevalence [29,30,42]. We did not directly measure the mechanisms, but we can frame several hypotheses for future research. One hypothesis is that hormonal differences, particularly the immunomodulatory effects of estrogen, contribute to a more reactive and thus more easily detectable inflammation-depression link in females [43]. Studies suggest that differences in gonadal hormone environments between males and females may influence antidepressant capacity and treatment efficacy in both sexes [43]. Additionally, compared to men, healthy women are more likely to exhibit depressive moods and social disengagement when facing stress [44], and their immune response to acute inflammation appears stronger [45]. Furthermore, sex differences in the transcriptional features of depression may fundamentally differ between females and males [29], with gender-specific morphological abnormalities observed in the cortical-limbic-striatal circuit in untreated depression patients [46]. Stress exposure or antidepressant treatment also has gender-specific effects on neurogenesis and dendritic remodeling in the adult hippocampus [29]. Another possibility is that the significant interactions in males reflect a greater vulnerability to the combined effects of inflammation and external stressors. For education, as a representative indicator of social determinants of health, males with lower education levels may be more susceptible to social role pressures and the mismatch of societal expectations, exhibiting poorer coping strategies and making them more prone to depressive symptoms [47–50]. In contrast, females may mitigate the negative psychological impact of lower educational attainment through a richer social support network or stronger emotional expression. Regarding alcohol consumption, chronic alcohol use can lead to increased intestinal permeability, gut microbiome imbalance, and upregulated expression of inflammatory factors, which intensify the impact of the inflammatory system on the central nervous system [51]. Males typically exhibit higher alcohol consumption frequency and dependence rates, and in those with elevated PIV levels, drinking behavior may further increase depression risk by enhancing immune activation effects [52–55]. The stronger association in younger men (<60) is particularly noteworthy and warrants further investigation, as it runs counter to typical age-related inflammation increases. Males are more likely to present a chronic low-grade inflammatory state during aging, as their immune systems gradually shift towards a pro-inflammatory direction, which may amplify the negative impact of high PIV levels on mood disorders [56–58]. In contrast, because estrogen inhibits inflammation, females may exhibit distinct inflammatory response trajectories even after menopause, weakening the interaction between age and PIV.

Several limitations must be acknowledged. First, the cross-sectional design precludes causal inference. Second, while we adjusted for many confounders, residual confounding from unmeasured variables like diet, sleep quality, or specific psychiatric comorbidities is possible. Third, our definition of probable depression was based on the PHQ-9, a screening tool rather than a clinical diagnosis. Fourth, the large number of subgroup and interaction analyses increases the risk of Type I error; these findings, particularly the interactions in males, should be considered exploratory and hypothesis-generating. Fifth, our study is based on a U.S. population, and findings may not be generalizable to other populations, especially in low- and middle-income countries where inflammatory and depression profiles differ. Finally, we used a complete-case analysis, which could introduce selection bias, although the excellent baseline balance mitigates this concern.

While our findings are potentially meaningful and provide preliminary evidence for the PIV-depression association, several important steps remain before PIV can be considered for clinical application in depression screening or diagnosis. The observed cutoff of 5.74 is exploratory and requires validation in independent, prospective cohorts to establish its reliability and generalizability across diverse populations and clinical settings. The primary added value of a biomarker like PIV would be to identify a subset of individuals with an inflammatory depression subtype, potentially guiding targeted anti-inflammatory therapies or personalized treatment strategies. However, this application requires further investigation. Future research should prioritize longitudinal studies to establish temporal relationships and causality, validation of PIV thresholds in diverse clinical populations, mechanistic studies to elucidate the biological pathways linking PIV to depression, and ultimately, intervention trials to determine whether modifying PIV levels can alter depression risk or improve treatment outcomes. These steps would help translate our findings into clinically actionable tools for depression prevention and management.

## Conclusion

In conclusion, higher PIV levels are associated with an increased risk of probable depression in the U.S. population, an association that is particularly robust in females. In males, this relationship is more complex and moderated by age, education, and alcohol use. PIV may be a promising, sex-specific biomarker for depression risk. However, substantial further research is required to validate these findings and explore the underlying mechanisms before any clinical utility can be considered.

## Supporting information

S1 Table The associations between PIV and probable depression. (DOCX)

S2 Table Gender differences in the associations between PIV and probable depression. (DOCX)
