# Supplementary material for: Gender differences in the association between pan-immune-inflammation value and probable depression: A cross-sectional study
Source: PLoS One. 2025 Dec 30;20(12):e0339348. doi: 10.1371/journal.pone.0339348 (PMC12752952; doi:10.1371/journal.pone.0339348)
Supplement: S1 Table — (DOCX) [file pone.0339348.s001.docx]

Supplementary Table S1 The associations between PIV and probable depression

| Character | Crude model | | Model 1 | | Model 2 | | Model 3 | |
| --- | --- | --- | --- | --- | --- | --- | --- | --- |
|  | OR(95%CI) | *P* | OR(95%CI) | *P* | OR(95%CI) | *P* | OR(95%CI) | *P* |
| Lower PIV | ref |  | ref |  | ref |  | ref |  |
| Higher PIV | 1.335 (1.164–1.531) | **<0.001** | 1.284 (1.119–1.474) | **<0.001** | 1.183 (1.026–1.364) | **0.021** | 1.154 (1.002–1.329) | **0.046** |
| PIV: pan-immune inflammation value; BMI: body mass index; PIR: poverty income ratio; CVD: cardiovascular disease; CKD: chronic kidney disease; Bold indicates *P* <0.05; ref: reference level/category.  Crude model: adjusted for none.  Model 1: adjusted for age, sex, race, education, PIR, smoke, alcohol, health insurance.  Model 2: Model 1 + adjusted for central obesity, BMI, and physical activity.  Model 3: Model 2 + adjusted for antidepressant use, diabetes, hypertension, CVD, CKD, cancer, and stroke. | | | | | | | | |
