# Supplementary material for: Gender differences in the association between pan-immune-inflammation value and probable depression: A cross-sectional study
Source: PLoS One. 2025 Dec 30;20(12):e0339348. doi: 10.1371/journal.pone.0339348 (PMC12752952; doi:10.1371/journal.pone.0339348)
Supplement: S2 Table — (DOCX) [file pone.0339348.s002.docx]

| Supplementary Table S2 Gender differences in the associations between PIV and probable depression   \| Character \| Crude model \| \| Model 1 \| \| Model 2 \| \| Model 3 \| \| \| --- \| --- \| --- \| --- \| --- \| --- \| --- \| --- \| --- \| \| OR(95%CI) \| *P* \| OR(95%CI) \| *P* \| OR(95%CI) \| *P* \| OR(95%CI) \| *P* \| \| Female \|  \|  \|  \|  \|  \|  \|  \|  \| \| PIV \| 1.29(1.14,1.46) \| **<0.001** \| 1.22(1.08,1.38) \| **0.0021** \| 1.15(1.02,1.30) \| **0.023** \| 1.13(1.00,1.27) \| 0.055 \| \| PIVcategory \|  \|  \|  \|  \|  \|  \|  \|  \| \| Lower PIV \| ref \|  \| ref \|  \| ref \|  \| ref \|  \| \| Higher PIV \| 1.41(1.21,1.63) \| **<0.001** \| 1.31(1.13,1.52) \| **<0.001** \| 1.24(1.07,1.44) \| **0.005** \| 1.20(1.03,1.40) \| **0.018** \| \| Male \|  \| \|  \| \|  \| \|  \| \| \| PIV \| 1.20(1.03,1.41) \| **0.021** \| 1.15(0.98,1.34) \| 0.086 \| 1.11(0.95,1.30) \| 0.179 \| 1.09(0.92,1.29) \| 0.323 \| \| PIVcategory \|  \|  \|  \|  \|  \|  \|  \|  \| \| Lower PIV \| ref \|  \| ref \|  \| ref \|  \| ref \|  \| \| Higher PIV \| 1.21(1.02,1.44) \| **0.032** \| 1.12(0.94,1.33) \| 0.217 \| 1.08(0.90,1.29) \| 0.398 \| 1.05(0.87,1.26) \| 0.622 \| \| PIV: pan-immune inflammation value; BMI: body mass index; PIR: poverty income ratio; CVD: cardiovascular disease; CKD: chronic kidney disease; Bold indicates P <0.05; ref: reference level/category.  Crude model: adjusted for none.  Model 1: Crude model + adjusted for age, sex, race, education, PIR, smoke, alcohol, health insurance.  Model 2: Model 1 + adjusted for central obesity, BMI, and physical activity.  Model 3: Model 2 + adjusted for antidepressant use, diabetes, hypertension, CVD, CKD, cancer, and stroke. \| \| \| \| \| \| \| \| \| |
| --- | --- | --- | --- | --- | --- | --- | --- | --- | --- | --- | --- | --- | --- | --- | --- | --- | --- | --- | --- | --- | --- | --- | --- | --- | --- | --- | --- | --- | --- | --- | --- | --- | --- | --- | --- | --- | --- | --- | --- | --- | --- | --- | --- | --- | --- | --- | --- | --- | --- | --- | --- | --- | --- | --- | --- | --- | --- | --- | --- | --- | --- | --- | --- | --- | --- | --- | --- | --- | --- | --- | --- | --- | --- | --- | --- | --- | --- | --- | --- | --- | --- | --- | --- | --- | --- | --- | --- | --- | --- | --- | --- | --- | --- | --- | --- | --- | --- | --- | --- | --- | --- | --- | --- | --- | --- | --- | --- | --- | --- | --- | --- | --- | --- | --- | --- | --- |
